# Evaluating the Validity of Risk Scoring in Predicting Pacemaker Rates following Transcatheter Aortic Valve Replacement

**DOI:** 10.1155/2020/1807909

**Published:** 2020-10-20

**Authors:** Alexander M. Spring, Michael A. Catalano, Vikram Prasad, Bruce Rutkin, Elana Koss, Alan Hartman, Pey-Jen Yu

**Affiliations:** Division of Cardiovascular and Thoracic Surgery, Zucker School of Medicine at Hofstra-Northwell, 300 Community Drive, 1DSU, Manhasset 11030, NY, USA

## Abstract

**Introduction:**

Requirement of permanent pacemaker (PPM) implantation is a known and common postoperative consequence of transcatheter aortic valve replacement (TAVR). The Emory risk score has been recently developed to help risk stratify the need for PPM insertion in patients undergoing TAVR with SAPIEN 3 valves. Our aim was to assess the validity of this risk score in our patient population, as well as its applicability to patients receiving self-expanding valves.

**Methods:**

We conducted a retrospective review of 479 TAVR patients without preoperative pacemakers from November 2016 through December 2018. Preoperative risk factors included in the Emory risk score were collected for each patient: preoperative QRS, preoperative right bundle branch block (RBBB), preoperative syncope, and degree of valve oversizing. Multivariable analysis of the individual variables within the scoring system to identify predictors of PPM placement was performed. The predictive discrimination of the risk score for the risk of PPM placement after TAVR was assessed with the area under the receiver operating characteristic curve (AUC).

**Results:**

Our results demonstrated that, of the 479 patients analyzed, 236 (49.3%) received balloon-expandable valves and 243 (50.7%) received self-expanding valves. Pacemaker rates were higher in patients receiving self-expanding valves than those receiving balloon-expandable valves (25.1% versus 16.1%, *p*=0.018). The Emory risk score showed a moderate correlation with pacemaker requirement in patients receiving each valve type, with AUC for balloon-expandable and self-expanding valves of 0.657 and 0.645, respectively. Of the four risk score components, preoperative RBBB was the only predictor of pacemaker requirement with an AUC of 0.615 for both balloon-expandable and self-expanding valves. *Conclusion. In our cohort*, the Emory risk score had modest predictive utility for PPM insertion after balloon-expandable and self-expanding TAVR. The risk score did not offer better discriminatory utility than that of preoperative RBBB alone. Understanding the determinants of PPM insertion after TAVR can better guide patient education and postoperative management.

## 1. Introduction

Transcatheter aortic valve replacement (TAVR) is now an established alternative to surgical aortic valve replacement for patients with severe aortic stenosis [1–4]. Despite its success and limited complication risk, the occurrence of conduction abnormalities and the need for permanent pacemaker (PPM) implantation remain the most frequent complication of TAVR [5].

Many studies have identified predictors of PPM implantation following TAVR [6–8]. Most recently, Kiani et al. developed the Emory risk score as a tool to aid in the risk stratification of patients undergoing TAVR with SAPIEN 3 balloon-expandable valves. The characteristics of the score include history of syncope, preexisting right bundle branch block (RBBB), QRS duration ≥140 ms, and valve oversizing ≥16% [9].

The aim of this study is to assess the validity of the Emory risk score in our patient population. Moreover, we sought to determine whether the model was applicable to both balloon-expandable and self-expanding valves.

## 2. Methods

This study was conducted with the approval of the Northwell Health System Institutional Review Board. As this is a retrospective study utilizing de-identified data collected from the New York State and STS databases, specific waiver of the need for individual patient consent was granted by the Institutional Review Board.

All patients who underwent TAVR for severe, symptomatic aortic stenosis from October 2016 to December 2018 were included in this study. All patients were implanted with either a Medtronic Evolut (Medtronic, Minneapolis, MN) or Edwards SAPIEN 3 (Edwards Lifesciences, Irvine, CA) valve. Patients with preexisting PPM or implanted cardiac defibrillators or those undergoing a valve-in-valve procedure were excluded. Preoperative characteristics included in the Emory risk score were collected for each patient, including preoperative QRS duration, preoperative RBBB, the presence of syncope as a symptom, and degree of valve oversizing. A risk score of 0–5 was calculated for each patient, with 1 point allocated for QRS ≥140 ms, syncope, and valve oversizing of ≥16%, and 2 points allocated for preoperative RBBB.

The following baseline preoperative data were also collected for each patient: age, gender, valve type, valve size, Society of Thoracic Surgery Predicted Risk of Mortality (STS-PROM), operator risk stratification, and other risk factors and comorbidities (i.e., dialysis, creatinine, cerebrovascular disease, peripheral artery disease, New York Heart Association heart failure class, diabetes, body mass index, and preoperative ejection fraction). The primary clinical endpoint of interest was the requirement of PPM post-TAVR.

Continuous variables are expressed as mean ± standard deviation and compared using Student's *t*-test. Categorical variables are expressed as percentages and compared using the chi-square test or Fisher's exact test, where appropriate. Differences in preoperative characteristics between patients who required PPM and those who did not were assessed. The association of each individual risk factor with the requirement of PPM was assessed using multivariable logistic regression analysis for both balloon-expandable and self-expanding valves. The accuracy of the Emory risk score and individual factors were assessed with area under receiver-operating characteristic (ROC) curve. Data analysis was performed retrospectively. All statistical analyses were performed using SAS Version 9.4 (SAS Institute Inc., Cary, NC).

## 3. Results

Preoperative characteristics and risk factors, including the components of the Emory risk score, are presented in Table 1. Of the 479 patients who underwent TAVR, 99 (20.7%) patients required PPM. Of the patients that underwent PPM, 86 (86.9%) patients required a pacemaker during the index TAVR admission, and 13 (13.1%) patients required a pacemaker following discharge. Among the entire cohort, 236 (49.3%) received balloon-expandable valves and 243 (50.7%) received self-expanding valves. Thirty-eight (16.1%) of patients receiving balloon-expandable valves required PPM, while 61 (25.1%) of patients receiving self-expanding valves required PPM (*p*=0.015).

The incidence of the elements included in the risk score among all patients was as follows: 12.3% of the patients had RBBB, 12.1% had a QRS duration ≥140 ms, 56% had valve oversizing ≥16%, and 3.8% had a history of syncope. Patients who required PPM post-TAVR were more likely to have preoperative RBBB (30.3% versus 7.7%, *p* < 0.001), had longer mean QRS duration (115.3 ± 27.6 versus 99.5 ± 23.7, *p* < 0.001), and were more likely to have a QRS duration ≥140 ms (23.2% versus 9.2%, *p* < 0.001). There was no significant difference in valve oversizing, presence of preoperative syncope, and other demographics and comorbidities between patients that received PPM versus those that did not. Patients who required PPM after TAVR had a higher Emory risk score as compared to those who did not require PPM (*p* < 0.001, Table 1).

The components of the Emory risk score assessed using multivariable analysis by valve type are presented in Table 2. Of the four risk score components, preoperative RBBB was the only independent predictor of pacemaker requirement, regardless of valve type. Among patients receiving balloon-expandable valves, 41.4% of patients were with RBBB-required pacemakers (OR 3.89, *p*=0.010); among those receiving self-expanding valves, 60.0% of patients were with RBBB-required pacemakers (OR 5.75, *p* < 0.001). Although QRS ≥140 ms was associated with PPM insertion after TAVR in the univariate analysis, it was no longer significant in the multivariable analysis for both valve types.

**Figure 1:**
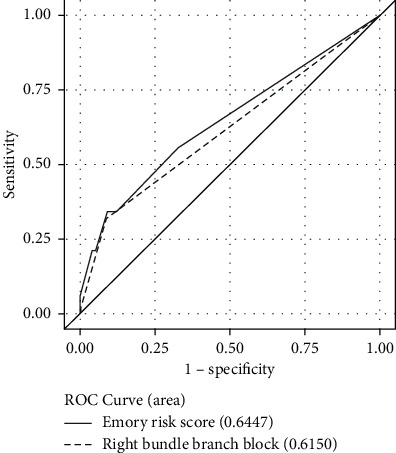
ROC curve for balloon-expandable valves.

**Figure 2:**
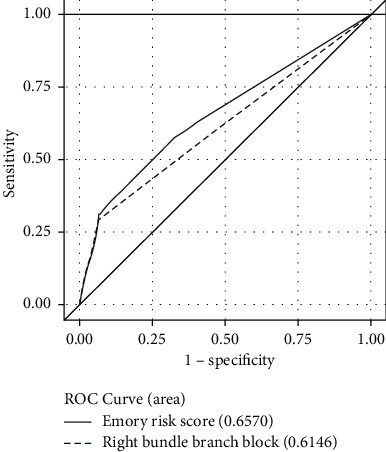
ROC curve for self-expanding valves.

The area under the ROC curve for the Emory risk score to discriminate for patients requiring PPM after TAVR was 0.645 for balloon-expandable valves (Figure 1) and 0.657 for self-expanding valves (Figure 2). The area under the ROC curve for the preoperative RBBB to discriminate for patients requiring PPM after TAVR was 0.615 for both balloon-expandable and self-expanding valves. The Emory risk score did not demonstrate significant superiority in discriminatory power over the presence of RBBB alone in predicting post-TAVR PPM requirements (*p*=0.350 for balloon-expandable valves and *p*=0.151 for self-expanding valves).

## 4. Discussion

Our results demonstrated that the Emory risk score, which stratifies patients based on QRS duration, preexisting RBBB, preoperative syncope, and valve oversizing have similar discriminatory ability for need for PPM after TAVR for balloon-expandable and self-expanding valves. The risk score, however, does not provide significantly increased discriminatory power over presence of preoperative RBBB alone.

The Emory risk score is the first contemporary scoring system to predict the need for PPM among patients undergoing TAVR [9]. It was developed by Kiani et al. and derived from data from a single institution undergoing Edwards SAPIEN 3 valves. It incorporates four characteristics: history of syncope, right bundle branch block, QRS duration ≥140 ms, and valve oversizing ≥16%. Kiani et al. reported an area under the curve for their Emory risk score of 0.778 in the validation cohort of patients undergoing SAPIEN 3 valves. Our study is the first to apply the Emory risk score to patients receiving Evolut balloon-expandable valves. While we found that the Emory risk score has similar discriminatory utility for risk of PPM after TAVR for both balloon-expandable and self-expanding valves, the area under the curve from our patient sample was significantly lower than that obtained by Kiani et al. (0.615 for both balloon-expandable valves and self-expanding valves). Differences in implant technique and institutional guidelines for PPM after TAVR may account for the differences in discriminatory utility of the risk score. This highlights the difficulty in developing universal risk scoring algorithms as algorithms developed in one institution may not be applicable to other institutions secondary to differences in practice patterns.

Incidence of elements of the risk score may vary by institution, further complicating the development of a universal algorithm. This is particularly true in elements of the risk score that are operator dependent. For instance, the incidence of valve oversizing ≥16% was substantially higher in our cohort relative to the Emory derivation cohort (56% versus 23.6%), highlighting likely differences in the valve type and size selection. Notwithstanding, studies have shown that >20% oversizing in self-expanding valves does not significantly increase the rate of PPM insertion [10]; thus, we do not believe that this variation would explain the differences in our outcomes. Further, the incidence of history of syncope was lower in our cohort (3.8% versus 9.4%). While lower than the Emory study, this remains consistent with the literature [11]. The incidence of RBBB (12.3% versus 15.6%) and QRS duration ≥140 ms (12.1% versus 13.6%) were comparable between our cohort and the derivation cohort in the Emory study.

In our sample, 20.7% of patients required PPM implantation after TAVR. In patients receiving balloon-expandable valves, the PPM rate was 16.1% versus 25.1% in self-expanding valves. The finding that PPM insertion rate is higher in patients receiving self-expanding valves is consistent with the literature. Previously published studies have shown the PPM rate to be as high as 17% for balloon-expandable valves [12] and 40% for self-expanding valves [13]. Preoperative RBBB was the only independent predictor of PPM implantation in our cohort, regardless of valve type. This is consistent with existing literature in which preoperative RBBB has been shown to be a well-described predictor of postoperative PPM implantation. In our study, preoperative RBBB offered similar discriminatory utility for need for PPM after TAVR as the Emory risk score [6, 14, 15]. While QRS duration was found to be a significant predictor of PPM on univariate analysis, there was no significance on multivariable analysis. This is likely due to the association between QRS duration and RBBB. Valve oversizing was not an independent predictor of PPM in our study, which is consistent with prior literature [16, 17], albeit not consistent with the Emory study. Similarly, while syncope is an independent predictor of need for PPM in the Emory risk score, we did not find it to be an independent predictor in our study. The low prevalence of syncope in our patient population may not have provided adequate statistical power to show significance.

There are other electrical, procedural, and anatomical factors that have been shown to be associated with an increased need for PPM after TAVR including first-degree heart block, implantation depth, length of the membranous septum, pre and postdilation of the prosthesis, and aortic annulus calcium score [18–22]. Our current study did not evaluate the association of such factors with PPM insertion as the primary objective of this study was to validate the Emory risk score which does not incorporate such factors.

There are several limitations to this study that should be acknowledged. First, there are no specific recommendations for PPM implantation after TAVR. Decisions to proceed with PPM may therefore be subject to selection bias. Second, while our overall sample size was large, the subset of patients who met specific criteria of the risk score was more limited. This may lead to type II error when evaluating the association of the specific criteria with requirement for PPM. However, the main objective of this study was to validate the Emory risk score, not the individual predictors of PPM placement. Third, although all clinical information relevant to the Emory risk score was independently validated for the purpose of this study, the study remains retrospective in nature and, therefore, has all the limitations of a retrospective study. Fourth, patients in this study received either SAPIEN 3 or Evolut valves. PPM implantation rate varies by both valve type and generation. The new-generation SAPIEN 3 valves have been associated with higher PPM implantation rates relative to the old-generation SAPIEN XT valves [23]. In contrast, the new-generation Evolut valves have lower PPM rates as compared with their first-generation counterparts [24]. As such, the results of this study may not be applicable to valve types and/or generations that are not utilized in our study population. Finally, as with all single-center studies, the results of this study may not be generalizable to other institutions. In fact, our finding that the Emory risk score displayed significantly less discriminatory utility in our patients as compared to its original validation cohort highlights this limitation.

## 5. Conclusions

In our cohort, the Emory risk score had modest predictive utility for PPM insertion after TAVR for both balloon-expandable and self-expanding prostheses. The risk score did not offer better discriminatory utility than that of preoperative RBBB alone.

## Figures and Tables

**Table 1:** Baseline patient characteristics: post-TAVR PPM versus no PPM.

Preoperative characteristics	No PPM	PPM	*p* value
	*N* = 380	*N* = 99	
Male	169 (44.6)	52 (52.5)	0.158
Age, years	82.3 ± 7.8	82.2 ± 10.5	0.960
Valve type			0.015
Self-expanding	182 (47.9)	61 (61.6)	—
Balloon-expandable	198 (52.1)	38 (38.4)	—
RBBB	29 (7.7)	30 (30.3)	<0.001
QRS duration	99.5 ± 23.7	115.3 ± 27.6	<0.001
QRS >140 ms	35 (9.2)	23 (23.2)	<0.001
Valve oversizing, %	12.91 ± 10.26	14.62 ± 9.83	0.136
Valve oversizing >16.0%	215 (56.6)	53 (53.5)	0.586
Syncope	13 (3.4)	5 (5.1)	0.447
Emory risk score			<0.001
Score = 0	178 (46.8)	32 (32.3)	—
Score = 1	159 (41.8)	32 (32.3)	—
Score = 2	23 (6.1)	12 (12.1)	—
Score = 3	14 (3.7)	15 (15.2)	—
Score = 4	6 (1.6)	8 (8.1)	—
Score = 5	0 (0.0)	0 (0.0)	—
STS-PROM, %	6.2 ± 6.1	6.3 ± 3.6	0.901
*Operator stratification*			0.692
Low risk	1 (0.3)	0 (0.0)	—
Intermediate risk	206 (54.2)	49 (50.0)	—
High risk	171 (45.0)	49 (50.0)	—
*Heart failure (NYHA)*			0.595
Class II	102 (26.9)	22 (22.2)	—
Class III	252 (66.5)	69 (69.7)	—
Class IV	25 (6.6)	8 (8.1)	—
Ejection fraction, %	61.8 ± 13.4	60.4 ± 13.5	0.352
Albumin, g/dL	3.9 ± 0.7	3.9 ± 0.7	0.942
Creatinine, mg/dL	1.3 ± 1.3	1.6 ± 1.7	0.079
Dialysis	13 (3.4)	4 (4.0)	0.766
Cerebrovascular disease	23 (6.1)	8 (8.1)	0.465
Peripheral artery disease	53 (13.9)	13 (13.1)	0.833
Diabetes	125 (32.9)	32 (32.3)	0.914
Body mass index	27.9 ± 5.9	28.4 ± 6.8	0.498

Continuous factors are given as mean (±standard deviation), compared using Student's *t*-test. Frequency and percent are given for categorical factors, compared using the chi-square test. NYHA = New York Heart Association; PPM = permanent pacemaker implantation; RBBB = right bundle branch block; STS-PROM = Society of Thoracic Surgeons Predicted Risk of Mortality.

**Table 2:** Multivariable analysis of predictors of postoperative PPM rates, by valve type.

Variable	Balloon-expandable		Self-expanding	
	OR (95% CI)	*p* value	OR (95% CI)	*p* value
Oversize >16%	1.58 (0.66, 3.83)	0.310	0.61 (0.32, 1.16)	0.134
Baseline RBBB	3.63 (1.31, 10.05)	0.013	5.57 (2.20, 14.10)	<0.001
Baseline QRS >140 ms	1.84 (0.62, 5.45)	0.270	1.15 (0.43, 3.06)	0.780
History of syncope	1.93 (0.42, 8.91)	0.400	1.36 (0.26, 7.07)	0.710

AUC of Emory risk score	0.645		0.657	

Odds ratios are given with 95% confidence interval. AUC = area under curve; PPM = permanent pacemaker implantation; RBBB = right bundle branch block.

## Data Availability

The data used to support the findings of this study are available from the corresponding author upon request.
